# Reviewer Acknowledgment

**DOI:** 10.1002/rmb2.12617

**Published:** 2024-12-26

The publication of invaluable papers in the Reproductive Medicine and Biology depends on the prompt careful review of submitted manuscripts. We would like to thank the Editorial Board members the Editorial Advisory Board members and the following experts for reviewing manuscripts from November 1, 2023, to October 31, 2024.

Shihoko Aizawa

Michiko Ammae

Yoshiyuki Asai

Tsuyoshi Baba

Adrià Costa‐Roig

Christopher Deibert

Noritoshi Enatsu

Kenji Ezoe

Shunsaku Fujii

Masakatsu Fujinoki

Maki Fukami

Rie Fukuhara

Shinichiro Fukuhara

Tatsuro Furui

Masataka Furuya

Robert B Gilchrist

Gürhan Güney

Jorge Hallak

Kenshiro Hara

Masayo Harada

Miyuki Harada

Hiroshi Harayama

Tomonari Hayama

Takahide Hayano

Ippei Hiramatsu

Yuji Hirao

Kenichiro Hiraoka

Toshitaka Horiuchi

Yuji Hotta

Shoko Ieda

Masashi Iijima

Kenji Imai

Hasan Inal

Tomohiro Ishii

Hiroshi Ishikawa

Tomomoto Ishikawa

Yuki Itani

Ayumu Ito

Junya Ito

Takuya Iwasawa

Shoichiro Iwatsuki

Takeshi Kajihara

Haruhiko Kanasaki

Satoru Kanto

Hanako Kaseki

Keiichi Kato

Kiyotaka Kawai

Kazuhiro Kawamura

Yasushi Kawano

Fuminori Kimura

Hiroshi Kishi

Yoshikazu Kitahara

Michio Kitajima

Kotaro Kitaya

Masato Kobanawa

Hideyuki Kobayashi

Ko Kobayashi

Tatsuya Kobayashi

Hiroshi Koike

Shahar Kol

Kouji Komatsu

Akira Komiya

Shinnosuke Komiya

Masataka Kudo

Sohei Kuribayashi

Keiji Kuroda

Shinnosuke Kuroda

Akari Kusamoto

Koichi Kyono

Ryo Maekawa

Tetsuo Maruyama

Mami Miyado

Shinichi Miyagawa

Satoshi Mizuno

Taisuke Mori

Tetsunori Mukaida

Ayako Muraoka

Aki Murashima

Masaji Nagaishi

Takeshi Nagamatsu

Tomoko Nakamura

Tatsuya Nakano

Yoshiharu Nakaoka

Akitoshi Nakashima

Masahiko Nakatsui

Yasuyuki Negishi

Yayoi Obata

Tsutomu Ogata

Kohei Ogawa

Shintaro Oka

Keisuke Okada

Asako Okamoto

Osamu Okitsu

Masanori Ono

Yosuke Ono

Aki Oride

Makoto Orisaka

Kuniaki Ota

Kazuki Saito

Manabu Satoh

Gaku Shimoi

Syogo Shiratsuki

Tetsuji Soda

Yukihiko Sugimoto

Koji Sugiura

Hironori Takahashi

Toshifumi Takahashi

Teppei Takeshima

Kentaro Takezawa

Isao Tamura

Kazuhiro Tamura

Kentaro Tanemura

Fuminori Taniguchi

Yuka Taniguchi

Hidetaka Tasaki

Hiroyuki Tomari

Shunichiro Tsuji

Sho Uchibori

Takashi Umehara

Mitsutoshi Yamada

Yuri Yamamoto

Kazumitsu Yamasaki

Yasuhisa Yamashita

Takayuki Yamochi

Kaoru Yanagida

Qi‐En Yang

Tsukasa Yoshida

Osamu Yoshino

Yasushi Yumura

